# RNA Inhibition Highlights Cyclin D1 as a Potential Therapeutic Target for Mantle Cell Lymphoma

**DOI:** 10.1371/journal.pone.0043343

**Published:** 2012-08-14

**Authors:** Shiri Weinstein, Rafi Emmanuel, Ashley M. Jacobi, Avigdor Abraham, Mark A. Behlke, Andrew G. Sprague, Tatiana I. Novobrantseva, Arnon Nagler, Dan Peer

**Affiliations:** 1 Laboratory of NanoMedicine, Department of Cell Research and Immunology, George S. Wise Faculty of Life Sciences, Tel Aviv University, Tel Aviv, Israel; 2 Center for Nanoscience and Nanotechnology, Tel Aviv University, Tel Aviv, Israel; 3 Integrated DNA Technologies, Inc., Coralville, Iowa, United States of America; 4 Division of Hematology, Chaim Sheba Medical Center, Tel Hashomer, Israel; 5 Alnylam Pharmaceuticals, Cambridge, Massachusetts, United States of America; University of Florida, United States of America

## Abstract

Mantle cell lymphoma is characterized by a genetic translocation results in aberrant overexpression of the CCND1 gene, which encodes cyclin D1. This protein functions as a regulator of the cell cycle progression, hence is considered to play an important role in the pathogenesis of the disease. In this study, we used RNA interference strategies to examine whether cyclin D1 might serve as a therapeutic target for mantle cell lymphoma. Knocking down cyclin D1 resulted in significant growth retardation, cell cycle arrest, and most importantly, induction of apoptosis. These results mark cyclin D1 as a target for mantle cell lymphoma and emphasize the therapeutic potential hidden in its silencing.

## Introduction

Mantle cell lymphoma (MCL) is an incurable B cell non-Hodgkin's lymphoma, characterized by the t(11; 14)(q13; q32) translocation that juxtaposes the proto-oncogene CCND1, which encodes cyclin D1 (cycD1), downstream of the immunoglobulin heavy chain gene promoter. This leads to overexpression of cycD1, which is not expressed in normal B cells [1]. cycD1 functions as an important regulator of the cell cycle G1-S transition. Complexes of cycD1 and cyclin-dependent kinase 4 (CDK4) or CDK6 phosphorylate retinoblastoma 1, thus leading to release of E2F transcription factors, which enable the subsequent progression of the cell into S phase [2], [3]. These complexes also titrate the CDK inhibitors p27kip1 and p21 away, therefore increase the kinase activity of cyclin E-CDK2 complexes, which enhance the transition into S phase [4].

The overexpression of cycD1, together with its established role as cell cycle progression regulator, highlight cycD1 as a potential central player in the pathogenesis of MCL [5]. However, a direct evidence for the therapeutic potential of manipulating this protein is still missing. Here we used RNA interference (RNAi) to potently down regulate cycD1 expression in well-characterized MCL cell lines. Using this strategy, we demonstrated that cycD1 could serve as a therapeutic target for MCL.

## Results and Discussion

**Figure 1:**
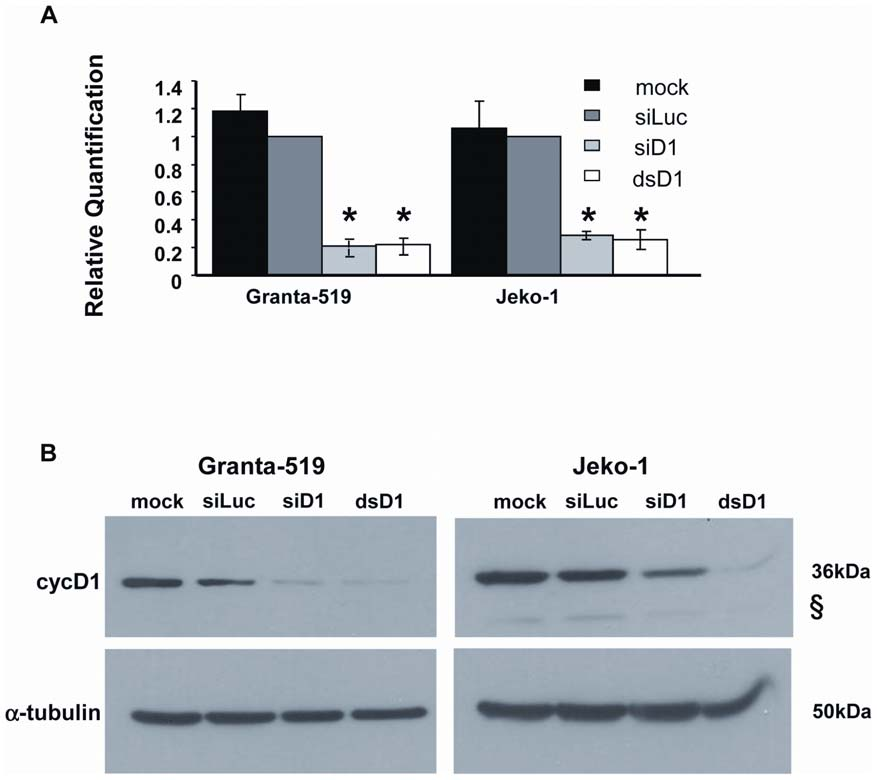
CCND1/cycD1 down regulation. (A) RT-qPCR analysis of CCND1 mRNA levels 48 h post electroporation in Granta-519 and Jeko-1 cells. Expression was normalized to both house keeping genes eIF3a and eIF3c and depicted as mRNA concentration relative to siLuc electroporated cells. Data are demonstrated as the mean ± SEM of three independent experiments. Significance strength was evaluated using one tailed ANOVA. * indicates p value <0.001. (B) Representative Western Blot analysis of cycD1 expression 48 h post electroporation. α-Tubulin was used as a loading control. § indicates cycD1b isoform.

**Figure 2:**
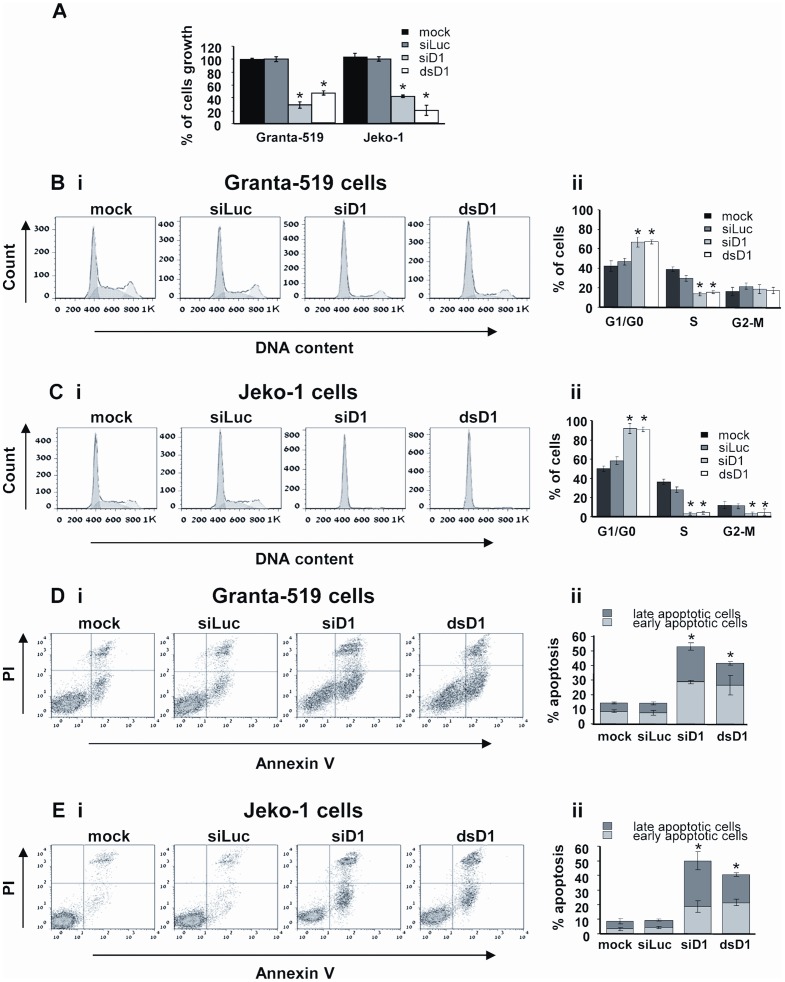
Effects of cycD1 down regulation on cell growth, cell cycle and apoptosis. (A) Proliferation rates of the untreated (mock), siLuc, siD1 and dsD1 electroporated Granta-519 and Jeko-1 cells as determined by the XTT cell proliferation assay, 7d post 1^st^ electroporation (5d post the 2^nd^ one). The results are demonstrated as percentage of cell growth in comparison to the siLuc electroporated cells. (B–C) Cell cycle distribution of mock, siLuc, siD1 and dsD1 electroporated Granta-519 (B) and Jeko-1 (C) cells, 72 h post 1^st^ electroporation (48 h post the 2^nd^ one). (i) Representative cell cycle histograms analysis, applied with the Dean-Jett-Fox model, using FlowJo™ software. (ii) Cell cycle distribution, demonstrated as percentage of cells in each phase. (D–E) apoptosis of mock, siLuc, siD1 and dsD1 electroporated Granta-519 (D) and Jeko-1 (E) cells, 8d post the 1^st^ electroporation (6d post the 2^nd^ one). (i) Representative dot-blot analysis using FlowJo™ software. Early apoptosis is demonstrated on cells labeled with Annexin V, while late apoptosis is demonstrated by Annexin V and PI labeled cells. (ii) Percentage of total apoptotic cells (divided into early and late fractions). All data are represented as the mean ± SEM of three independent experiments. Significance was evaluated using one tailed ANOVA. * indicates p value <0.001.

In this study, we aimed to validate cycD1 as a therapeutic target for MCL. We down regulated cycD1 in two model cell lines: Granta-519 and Jeko-1. Electroporating these cells with two different RNAi strategies, cycD1-siRNA (siD1) and cycD1-dicer substrate (dsD1), which were designed to specifically target different regions of the CCND1 mRNA, resulted in significant decrease in CCND1 mRNA and cycD1 protein levels (Figure 1). To maintain the low expression levels of cycD1 we preformed a 2^nd^ electroporation 48 h post the first one, which did not significantly effect the cells viability (Figures S1, S2). The reduction in cycD1 expression was followed by a major retardation in cell proliferation rates (Figure 2A) and G0/G1 phase cell arrest (Figures 2B–C, S3, S4). Most importantly, 7-10*d* post electroporation with siD1 or dsD1, Granta-519 and Jeko-1 cells exhibited a significant increase in the percentage of apoptotic cells (Figure 2D–E, S5). These results highlight cycD1 as a potential therapeutic target for MCL.

Manipulating the expression of cycD1 in MCL cells, using different strategies and compounds, has been shown previously to reduce cells' viability and survival indexes [6], [7], [8], [9]. However, all of these compounds modulate the expression of other factors known to be essential for cells viability, questioning the contribution of cycD1 down regulation. Here, exploiting the gene-specificity advantage of the RNAi approaches, we demonstrated for the first time that selective down regulation of cycD1 expression, is an adequate strategy for impairing MCL cells viability.

The therapeutic potential of cycD1 in MCL was revealed due to the rapid and potent silencing achieved with our screened RNAi sequences. It is worth mentioning that the relevance of selective cycD1 silencing has been questioned before [10], [11]. These previous studies demonstrated that down regulation of the sole cycD1 in MCL cells reduces proliferation rates and induces cell cycle arrest but is not sufficient for impairing MCL cells viability. The significant difference seen in the present study following cycD1 knockdown may be explained by the RNAi tools employed here to suppress cycD1 expression; siRNA and dicer substrate RNA (DsiRNA). Using these highly screened and selective RNAi strategies resulted in onset of very potent silencing as early as 48 *h* post electroporation. Kiler *et al.* [10] and Tchakarska *et al*. [11] used two shRNA approaches, both required more processing steps at the transcription and maturation stages in comparison to siRNA or DsiRNA that are directly incorporated into the RNA-induced silencing complex [12] or the Dicer enzyme [13], respectively. As a result, with the shRNA strategies, maximal silencing was achieved only about 5*d* later than with the siRNA/DsiRNA strategies. Since with the shRNA strategies, cell death was evaluated at the same day of maximal silencing, and the apoptosis in our study appeared relatively late (started at 7*d* post electroporation, or 5*d* post maximal silencing), it is reasonable to assume that later evaluation would result in significant apoptosis using the shRNA strategies as well.

The therapeutic advantage of silencing cycD1 in MCL is emphasized when taken into consideration the results presented in this study together with two recently published studies [14], [15], in which sensitization of MCL cells to different therapeutic agents following cycD1 down regulation is demonstrated. We reason that the manipulation of cycD1 expression opens two different battlefronts against the malignant cells; the first sensitizes the cells to apoptosis inducers, and the later, that is demonstrated in this study for the first time, origins from the cells response to cycD1 silencing per se. Further studies should address the question if the late cell death following cycD1 silencing could eliminate the chemotherapeutic resistant MCL cells, thus possibly reducing patient relapse rates, which is one of the most challenging obstacles in the treatment of MCL.

## Materials and Methods

### Cell culture

Granta-519 and Jeko-1 cells were purchased from the American Type Culture Collection (ATCC) and cultured as recommended.

### siRNA and Dicer-substrate sequences

siRNA sequences against the CCND1 gene NM_053056 (siD1, sense strand: GUAGGACUCUCAUUCGGGATT) and the luciferase gene as a control sequence (siLuc, sense strand: CUUACGCUGAGUACUUCGA) were designed and screened by Alnylam Pharmaceuticals (Cambridge MA, USA). Dicer-substrate (DsiRNA) against the CCND1 gene (dsD1, sense strand: GGAGCAUUUUGAUACCAGAAGGGAA) was designed and screened by Integrated DNA Technologies (IDT, Coralville, IA, USA). The screening was based on silencing efficacy and specificity.

### Electroporation

1 nmole of each of the RNA duplexes (siLuc/siD1/dsD1) were electroporated into 10x10^6^ Granta-519 or Jeko-1 cells using the Amaxa 4D-nuclefactor system (CM-119 program, SF solution). To maintain the expression of cycD1 low for longer period of time, a 2^nd^ electroporation was preformed 48 *h* post the first one.

### Quantitative real-time PCR

Total RNA was isolated using EZ-RNA kit (biological industries, Israel) and cDNA was generated with high capacity cDNA kit (Life Technologies, Carlsbad, CA, USA) according to the manufacturers' protocols. qRT-PCR was performed with Fast SYBR® Green Master Mix and the ABI StepOnePlus^TM^ instrument (Life Technologies). CCND1 (F:GAGGAGCCCCAACAACTTC C, R:GTCCGGGTCACACTTGATCAC) expression was normalized to the house keeping genes eIF3a (F:TCCAGAGAGCCAGTCCATGC, R:CCTGCCACAATTCA TGCT) and eIF3c (F:ACCAAGAGAGTTGTCCGCAGTG, R:TCATGGCATTACG GATGGTCC). Analysis was done with the StepOne^TM^ software V 2.1 (Life Technologies) using the multiple endogenous controls option. When using multiple endogenous controls, the software treats all endogenous controls as a single population, and calculates the experiment-appropriate mean to establish a single value against which the target of interest is normalized.

### Western blot analysis

Western blots were performed as described previously [16]. For protein detection, mouse monoclonal antibodies, anti human cycD1 (lifespan biosciences) and anti human α–tubulin (sigma), and a secondary antibody, goat anti mouse IgG horseradish peroxidase (sigma), were used.

### Cell proliferation

Following a 2^nd^ electroporation, cells were seeded onto 96 wells plate (1×10^5^ cells per a well, 6 wells for a condition). *5d* later, cell proliferation was measured using the cell proliferation kit (XTT, biological industries) as recommended by the manufacturer.

### Cell cycle and apoptosis studies

For cell cycle analysis, 5×10^5^ cells were collected 72 *h* post the first electroporation (24 h post the 2^nd^ one). The cells were washed with ice-cold PBS, and fixed with 70% ethanol for 1 *h*. Then, the cells were washed twice with cold PBS and incubated for 10 min at 37°C in 300 μL PBS with 15 μg/mL propidium iodide (PI) and 2.5 μg/mL DNase-free RNase A (Sigma, St. Louis, MO, USA). Fluorescence was measured by flow cytometry. Apoptosis was evaluated by flow cytometry using Annexin V and PI apoptosis detection kit (eBioScience, San Diego, CA, USA) as recommended by the manufacturer. Data from at least 10^4^ cells were acquired using BD FACScalibur™ and the CellQuest™ software. Analyses were done with FlowJo™ software. For cell cycle analysis, the Dean-Jett-Fox model was applied on at least 10,000 gated cells.

### Statistical analysis

The results are represented as the mean ± SEM of three independent experiments. One tailed ANOVA was preformed to estimate the significance strength of the results.

## Supporting Information

Figure S1 **A 2^nd^ electroporation is essential to maintain CCND1 expression low in MCL cell lines.** (A+C) RT-qPCR analysis of CCND1 mRNA levels in Granta-519 (A) and Jeko-1 (C) cells, 24 h – 8d post a single electroporation. (B+D) RT-qPCR analysis of CCND1 mRNA levels in Granta-519 (B) and Jeko-1 (D) cells underwent a 2^nd^ electroporation 48 h post the first one. Expression was normalized to both house keeping genes eIF3a and eIF3c and depicted as mRNA concentration relative to siLuc electroporated cells. Data are demonstrated as the mean ± SEM of three independent experiments.
(TIF)

Figure S2 **Effects of 2^nd^ electroporation on MCL cell lines viability.** Early and late apoptosis rates of mock, siLuc, siD1 and dsD1 electroporated Granta-519 and Jeko-1 cells were determined using double staining of Annexin V and PI. (A) Granta-519 cells, 72 h post a single electroporation. (B) Granta-519 cells, 24 h post the 2^nd^ electroporation preformed 48 h after the first one. (C) Jeko-1 cells, 72 h post a single electroporation. (D) Jeko-1 cells, 24 h post the 2^nd^ electroporation preformed 48 h after the first one. Representative dot-blot analysis of each condition is demonstrated. Above each dot blot, the apoptosis rates are represented as the mean ± SD of three independent experiments.
(TIF)

Figure S3 **The effects of 2^nd^ electroporation on the cell cycle distribution of Granta-519 cells.** Representative cell cycle histograms of mock, siLuc, siD1 and dsD1 electroporated Granta-519 cells applied with the Dean-Jett-Fox model, using FlowJo™ software. (A) 72 h post a single electroporation. (B) 24 h post a 2^nd^ electroporation performed 48 h post the first one. (C) 7d post a single electroporation. (D) 5d post a 2^nd^ electroporation preformed 48 h post the first one. Percentage of cells ± SD in each cell cycle phase is represented above the histograms.
(TIF)

Figure S4 **The effects of 2^nd^ electroporation on the cell cycle distribution of Jeko-1 cells.** Representative cell cycle histograms of mock, siLuc, siD1 and dsD1 electroporated Jeko-1 cells applied with the Dean-Jett-Fox model, using FlowJo™ software. (A) 72 h post a single electroporation. (B) 24 h post a 2^nd^ electroporation performed 48 h post the first one. (C) 7d post a single electroporation. (D) 5d post a 2^nd^ electroporation preformed 48 h post the first one. Percentage of cells ± SD in each cell cycle phase is represented above the histograms.
(TIF)

Figure S5 **A 2^nd^ electroporation of siD1/dsD1 enhanced the cell death rates of MCL cell lines.** Early and late apoptosis rates of mock, siLuc, siD1 and dsD1 electroporated Granta-519 and Jeko-1 cells were determined using double staining of Annexin V and PI. (A) Granta-519 cells, 8d post a single electroporation. (B) Granta-519 cells, 6d post a 2^nd^ electroporation preformed 48 h after the first one. (C) Jeko-1 cells, 8d post a single electroporation. (D) Jeko-1 cells, 6d post a 2^nd^ electroporation preformed 48 h after the first one. Representative dot-blot analysis of each condition is demonstrated. Above each dot blot, the apoptosis rates are represented as the mean ± SD of three independent experiments.
(TIF)
