# Supplementary figures and images for: RNA Inhibition Highlights Cyclin D1 as a Potential Therapeutic Target for Mantle Cell Lymphoma
Source: PLoS One. 2012 Aug 14;7(8):e43343. doi: 10.1371/journal.pone.0043343 (PMC3419170; doi:10.1371/journal.pone.0043343)

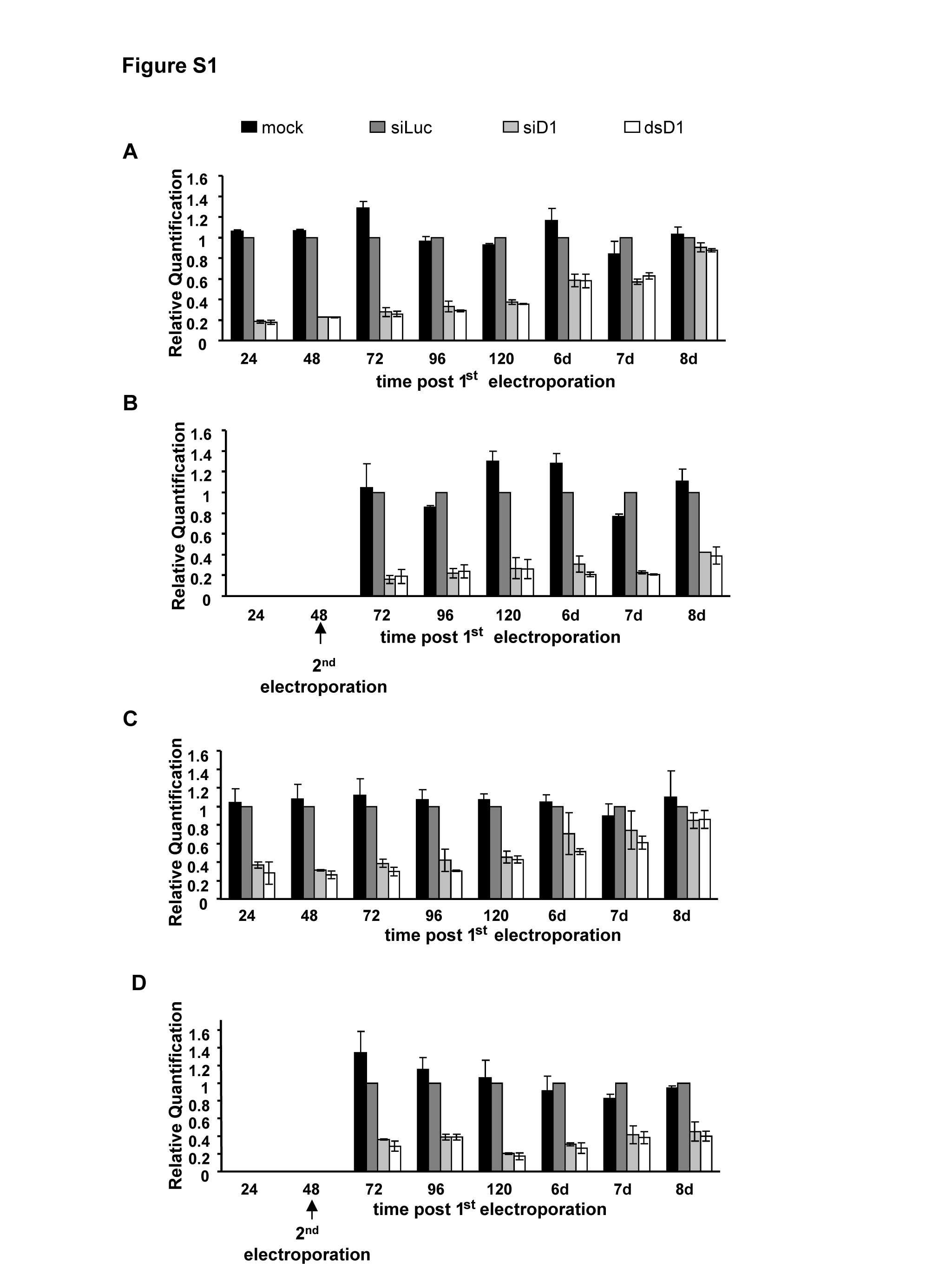

Supplement: Figure S1 — A 2nd electroporation is essential to maintain CCND1 expression low in MCL cell lines. (A+C) RT-qPCR analysis of CCND1 mRNA levels in Granta-519 (A) and Jeko-1 (C) cells, 24 h – 8d post a single electroporation. (B+D) RT-qPCR analysis of CCND1 mRNA levels in Granta-519 (B) and Jeko-1 (D) cells underwent a 2nd electroporation 48 h post the first one. Expression was normalized to both house keeping genes eIF3a and eIF3c and depicted as mRNA concentration relative to siLuc electroporated cells. Data are demonstrated as the mean ± SEM of three independent experiments. (TIF) [file pone.0043343.s001.tif]

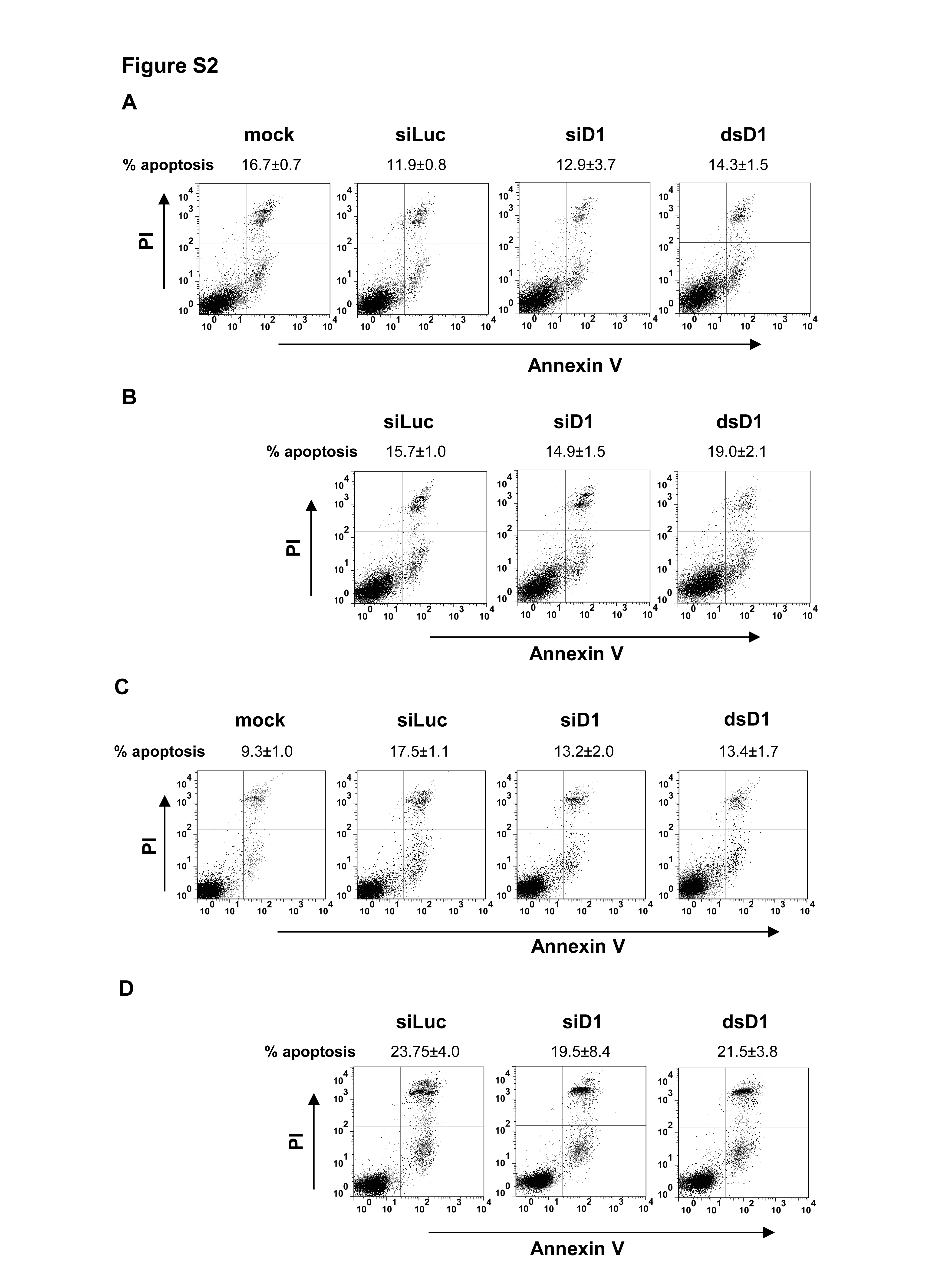

Supplement: Figure S2 — Effects of 2nd electroporation on MCL cell lines viability. Early and late apoptosis rates of mock, siLuc, siD1 and dsD1 electroporated Granta-519 and Jeko-1 cells were determined using double staining of Annexin V and PI. (A) Granta-519 cells, 72 h post a single electroporation. (B) Granta-519 cells, 24 h post the 2nd electroporation preformed 48 h after the first one. (C) Jeko-1 cells, 72 h post a single electroporation. (D) Jeko-1 cells, 24 h post the 2nd electroporation preformed 48 h after the first one. Representative dot-blot analysis of each condition is demonstrated. Above each dot blot, the apoptosis rates are represented as the mean ± SD of three independent experiments. (TIF) [file pone.0043343.s002.tif]

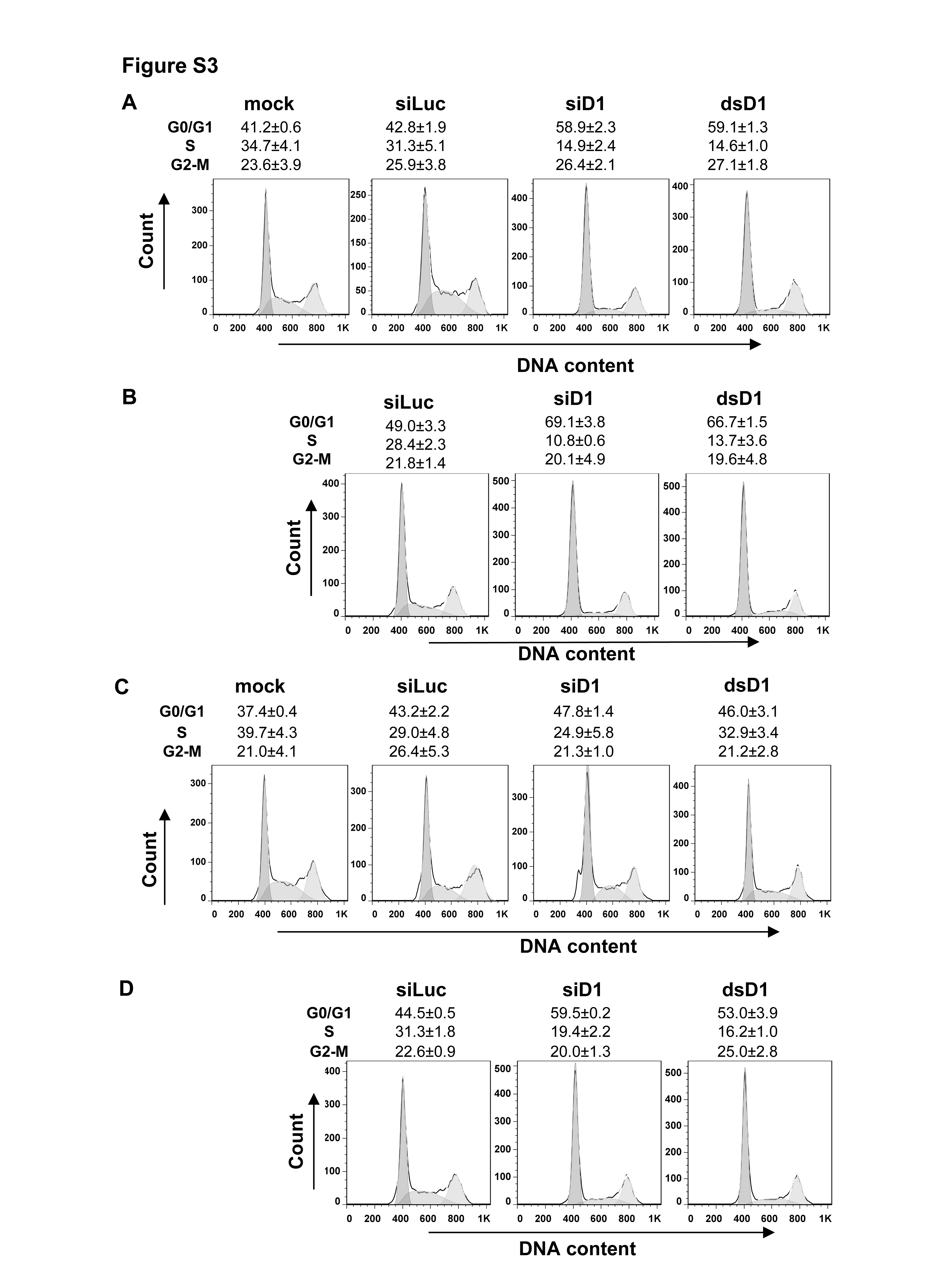

Supplement: Figure S3 — The effects of 2nd electroporation on the cell cycle distribution of Granta-519 cells. Representative cell cycle histograms of mock, siLuc, siD1 and dsD1 electroporated Granta-519 cells applied with the Dean-Jett-Fox model, using FlowJo™ software. (A) 72 h post a single electroporation. (B) 24 h post a 2nd electroporation performed 48 h post the first one. (C) 7d post a single electroporation. (D) 5d post a 2nd electroporation preformed 48 h post the first one. Percentage of cells ± SD in each cell cycle phase is represented above the histograms. (TIF) [file pone.0043343.s003.tif]

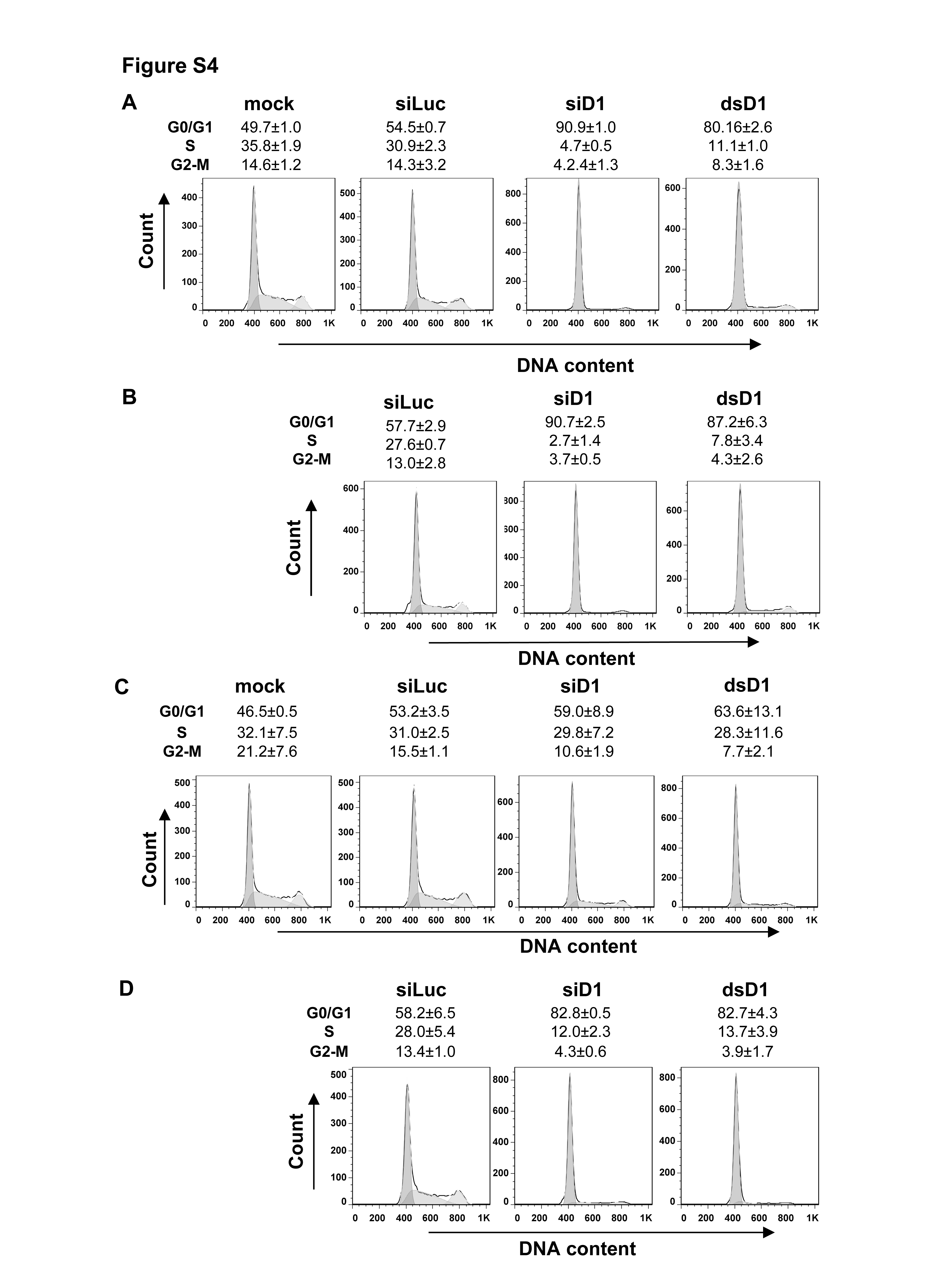

Supplement: Figure S4 — The effects of 2nd electroporation on the cell cycle distribution of Jeko-1 cells. Representative cell cycle histograms of mock, siLuc, siD1 and dsD1 electroporated Jeko-1 cells applied with the Dean-Jett-Fox model, using FlowJo™ software. (A) 72 h post a single electroporation. (B) 24 h post a 2nd electroporation performed 48 h post the first one. (C) 7d post a single electroporation. (D) 5d post a 2nd electroporation preformed 48 h post the first one. Percentage of cells ± SD in each cell cycle phase is represented above the histograms. (TIF) [file pone.0043343.s004.tif]

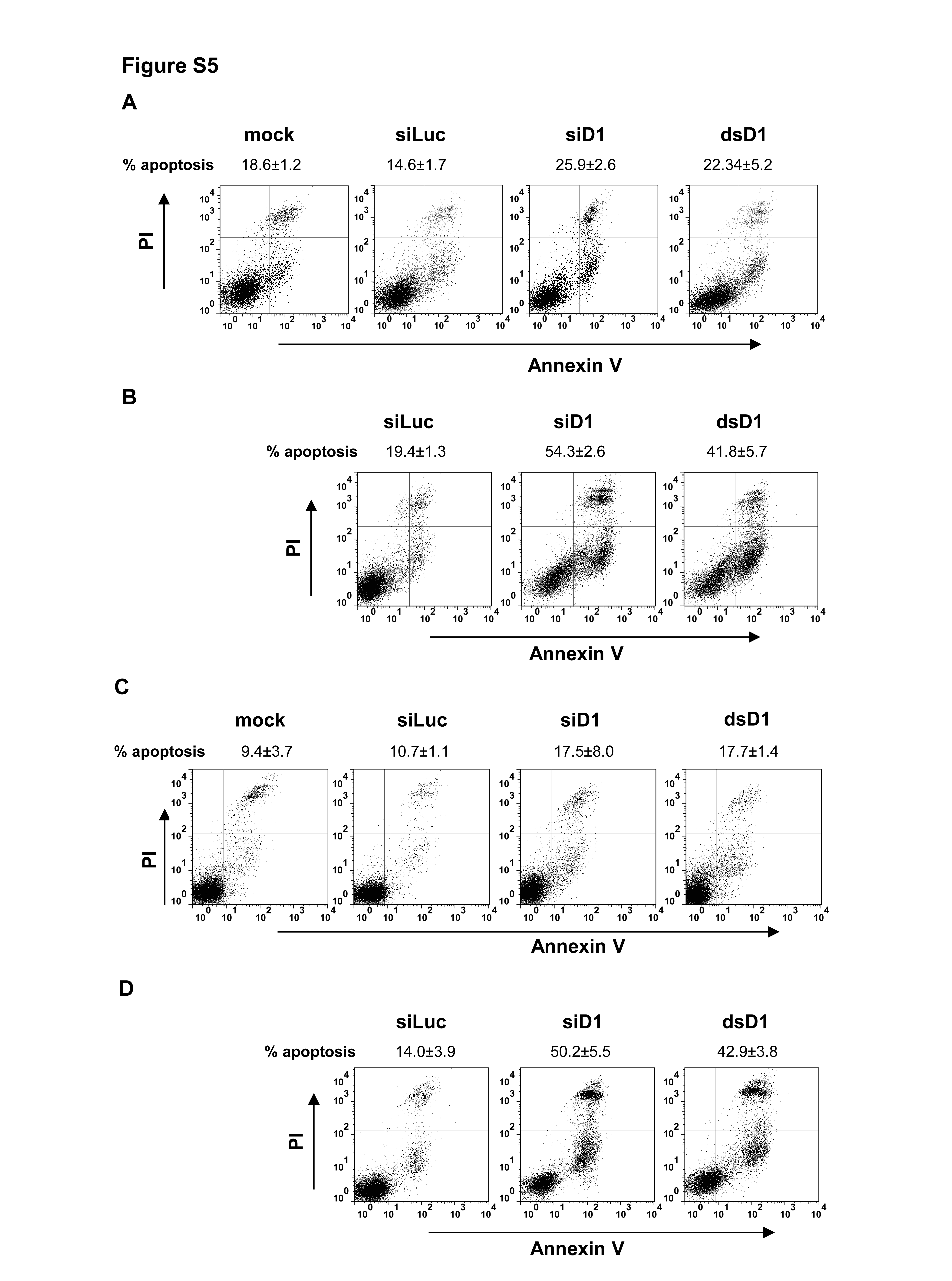

Supplement: Figure S5 — A 2nd electroporation of siD1/dsD1 enhanced the cell death rates of MCL cell lines. Early and late apoptosis rates of mock, siLuc, siD1 and dsD1 electroporated Granta-519 and Jeko-1 cells were determined using double staining of Annexin V and PI. (A) Granta-519 cells, 8d post a single electroporation. (B) Granta-519 cells, 6d post a 2nd electroporation preformed 48 h after the first one. (C) Jeko-1 cells, 8d post a single electroporation. (D) Jeko-1 cells, 6d post a 2nd electroporation preformed 48 h after the first one. Representative dot-blot analysis of each condition is demonstrated. Above each dot blot, the apoptosis rates are represented as the mean ± SD of three independent experiments. (TIF) [file pone.0043343.s005.tif]
